# Transcriptome Profiling Provides Molecular Insights into Auxin-Induced Adventitious Root Formation in Sugarcane (*Saccharum* spp. Interspecific Hybrids) Microshoots

**DOI:** 10.3390/plants9080931

**Published:** 2020-07-23

**Authors:** Aomei Li, Prakash Lakshmanan, Weizhong He, Hongwei Tan, Limin Liu, Hongjian Liu, Junxian Liu, Dongliang Huang, Zhongliang Chen

**Affiliations:** 1 Key Laboratory of Sugarcane Biotechnology and Genetic Improvement (Guangxi), Ministry of Agriculture and Rural affairs/Guangxi Key Laboratory of Sugarcane Genetic Improvement/Sugarcane Research Institute, Guangxi Academy of Agricultural Sciences, Nanning 530007, China; liaomei1995@sina.com (A.L.); plakshmanan2018@outlook.com (P.L.); 201liuliling@163.com (L.L.); lhj@gxaas.net (H.L.); liujunxian868@163.com (J.L.); Hdl666@163.com (D.H.); czl_good2007@163.com (Z.C.); 2 Interdisciplinary Research Center for Agriculture Green Development in Yangtze River Basin (CAGD), College of Resources and Environment, Southwest University, Chongqing 400715, China; 3 Queensland Alliance for Agriculture and Food Innovation, The University of Queensland, St Lucia 4072, QLD, Australia

**Keywords:** sugarcane, auxin, transcriptomics, adventitious root formation

## Abstract

Adventitious root (AR) formation was enhanced following the treatment of sugarcane microshoots with indole-3-butyric acid (IBA) and 1-naphthalene acetic acid (NAA) combined, suggesting that auxin is a positive regulator of sugarcane microshoot AR formation. The transcriptome profile identified 1737 and 1268 differentially expressed genes (DEGs) in the basal tissues (5 mm) of sugarcane microshoots treated with IBA+NAA compared to nontreated control on the 3rd and 7th days post-auxin or water treatment (days post-treatment—dpt), respectively. To understand the molecular changes, Gene Ontology (GO) and Kyoto Encyclopedia of Genes and Genomes (KEGG) analyses were performed. This analysis showed that DEGs associated with the pathways were associated with plant hormone signaling, flavonoid and phenylpropanoid biosyntheses, cell cycle, and cell wall modification, and transcription factors could be involved in sugarcane microshoot AR formation. Furthermore, qRT–PCR analysis was used to validate the expression patterns of nine genes associated with root formation and growth, and the results were consistent with the RNA-seq results. Finally, a hypothetical hormonal regulatory working model of sugarcane microshoot AR formation is proposed. Our results provide valuable insights into the molecular processes associated with auxin-induced AR formation in sugarcane.

## 1. Introduction

Sugarcane (*Saccharum* spp. interspecific hybrids), a major industrial crop, contributes approximately 80% of global sugar production with an annual value of USD150 billion [1]. Sugarcane has a large, complex genome due to its high and variable chromosome number and polyploidy [2]. Sugarcane is propagated vegetatively for commercial crop production [3]. Historically, nodal cuttings of sugarcane stem, called billets, or the entire stem were used for planting commercial crops. The traditional method of propagating sugarcane planting material in the field has increasingly been replaced by micropropagation. Micropropagation has the advantages of rapid production and scale-up potential for supplying diverse varieties to meet seasonal and regional demand, round-the-year supply, and easy transportation. It also requires a very small production area relative to field propagation and the provision of pest- and disease-free planting material, which is extremely important for maintaining maximum crop yield and biosecurity of the sugarcane industry [4]. In addition, micropropagation is increasingly used for accelerated adoption of new releases from most of the major sugarcane breeding programs globally. Micropropagation is widely used in horticulture, floriculture, forestry, and agriculture for the production of true-to-type, disease-free planting material [5]. Generally, shoots are produced from pathogen-free shoot meristems or shoot buds and multiplied under sterile in-vitro conditions, and they are rooted in vitro or ex vitro, depending on the species. For many woody and perennial species, production of roots from shoots, called AR formation, is a difficult and propagation-productivity-limiting step [6]. Hence, there is much commercial and research interest in AR formation.

Research to-date indicates a coordinated regulation of AR formation by phytohormones and many other endogenous and external stimuli [7]. Auxin phytohormones, alone or by interacting with ethylene (ET), cytokinin (CTK), salicylic acid (SA), brassinosteroid (BR), and jasmonic acid (JA), regulate root development [8]. Auxins, particularly indole-3-butyric acid (IBA) and 1-naphthalene acetic acid (NAA), are usually applied exogenously, alone or in combination, to promote AR formation [9]. Many studies on AR to-date were conducted to evaluate the effectiveness of various plant growth substances and growing conditions for rooting, as well as to develop practical and useful methods for rooting [10]. In general, research on the genetic or molecular basis of AR formation has been carried out in model plant species like *Arabidopsis*. Early steps in AR formation involve the establishment of the IAA gradient and its accumulation in specific cell types via polar auxin transport (PAT) and local auxin conjugation [11]. A defect in PAT leads to a reduction of AR formation via the inhibition or modification of founder cell priming and cell division [11]. The ATP-BINDING CASSETTE B19 (ABCB19) seems to be the main IAA efflux carrier involved in auxin accumulation, and it thus contributes to AR formation [7]. GRECBEN HAGEN3 (GH3) genes are involved in auxin conjugation, which would be required for fine-tuning adventitious root initiation in the *Arabidopsis* thaliana hypocotyl [12]. Small AUXIN UP RNAS Like (SAUR-like) proteins also play a role in induction and differentiation in excision-induced AR formation [13]. The auxin response factors AUXIN RESPONSE FACTOR 6 (ARF6) and ARF8 have been reported as positive regulators of AR formation, while ARF17 has an opposite effect [14]. In the presence of auxin, TIR1 and AFB2 form specific sensing complexes with IAA6, IAA9, and/or IAA17 that modulate JA homeostasis to control AR initiation [15]. Jasmonate-induced ETHYLENE RESPONSE FACTOR (ERF) 115 activates the cytokinin signaling machinery and thereby represses adventitious rooting in *Arabidopsis* [16]. Several AP2/ERF genes have been recently identified as potential players in tissue regeneration, and they may have a critical role in AR formation [7]. Auxin application strongly modified the expression of 40 genes related to cell wall weakening and modification [13]. Additionally, the functional role of enzymes controlling cell wall remodeling during lateral root development has been reported in *Arabidopsis* [13]. Cyclin-dependent kinases and cyclins are involved in the auxin-mediated regulation of the cell cycle [13]. The RETINOBLASTOMA-RELATED (RBR) gene is considered a key cell-cycle regulator downstream of the SCARECROW (SCR) patterning gene, controlling cell division, differentiation, and cell homeostasis. Auxin-responsive transcription factor SCR has also been shown to play important regulatory roles in cell division and root primordia differentiation [12]. Transcription factors (TFs) of the GRAS family, such as SCARECROW (SCR), are important regulators of root development [13].

Recent transcriptome analysis has correlated hormone signal transduction, carbohydrate and energy metabolisms, protein degradation, and certain transcription factor (TF) activities with AR formation in *Taxodium* “Zhongshanshan” [17]. A relationship between adventitious rooting, with or without IBA, and antioxidant enzymes was observed in mung bean seedlings [18]. IBA-induced AR formation was associated with indole-3-acetic acid (IAA) biosynthesis and transport in *Arabidopsis thaliana* [19]. Kang et al. reported endogenous hormone production, nitric oxide (NO), hydrogen peroxide (H_2_O_2_) and auxin signaling, and cell wall modification during AR formation in tea plants [20]. There is a large variation for AR formation in different sugarcane genotypes. However, little is known about the physiological or molecular aspects of adventitious rooting in sugarcane.

In the present study, the effect of IBA and NAA on AR formation and the gene expression occurring during adventitious rooting in micropropagated sugarcane shoots (microshoots) under photoautotrophic condition is investigated. Because IBA and NAA are routinely used for AR formation in many plant species, we conducted a series of preliminary experiments to determine the optimum auxin regimes for sugarcane microshoot AR formation. Auxin IBA at 50 mg/L combined with NAA at 20 mg/L gave the best results for AR formation (data not shown and not be published). The basal stem of microshoots treated with 50 mg/L IBA and 20 mg/L NAA was used for the transcriptome analysis. This is the first study of genes associated with AR formation in sugarcane microshoots grown under photoautotrophic conditions [21]. Since there is no fully annotated high-quality reference genome published for commercial sugarcane varieties, the de-novo assembly of Illumina short reads was conducted in this study. It allowed the identification of genes associated with AR formation in sugarcane, thus advancing our knowledge on the complex mechanism underpinning auxin-induced AR formation, a developmental process little explored from a molecular perspective.

## 2. Results

### 2.1. A Combination of IBA and NAA Increases the Number and Length of Adventitious Roots

**Figure 1:**
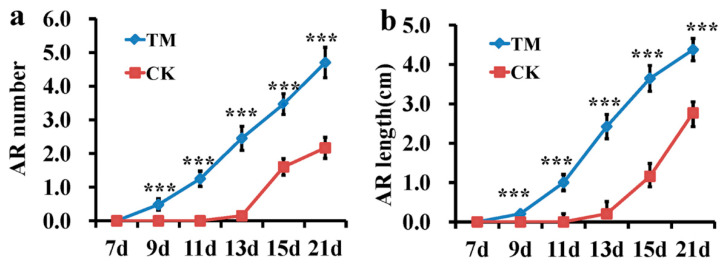

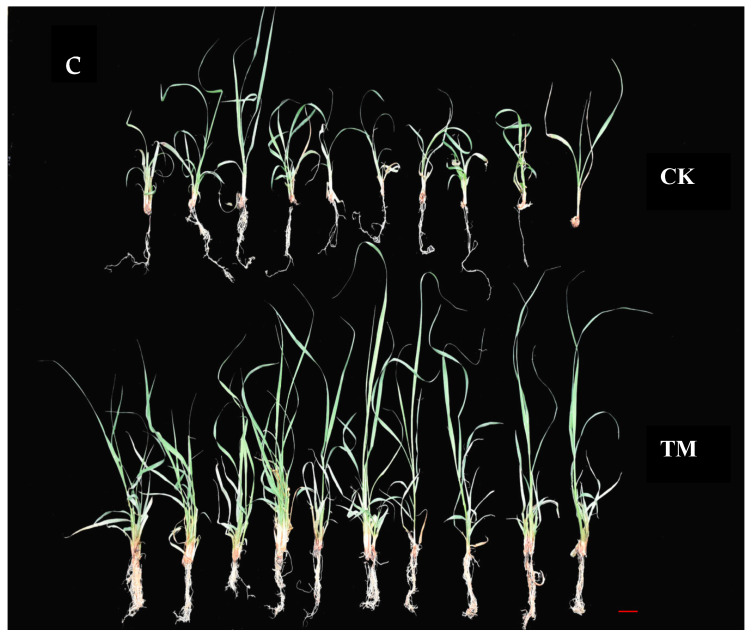
Effects of the indole-3-butyric acid (IBA) and 1-naphthalene acetic acid (NAA) mixture (auxin) on adventitious root (AR) formation in sugarcane. (**a**) Root number in sugarcane with auxin treatment and the control at 7, 9, 11, 13, 15, and 21 dpt (days post-treatment), *** are significantly different (*p* ≤ 0.01) (Independent-Samples *t*-test), data was presented as mean (±SE). (**b**) Root length in sugarcane following auxin treatment and the control at 7, 9, 11, 13, 15, and 21 dpt, *** are significantly different (*p* ≤ 0.01) (Independent-Samples *t*-test), data was presented as mean (±SE). (**c**) Morphology of microshoots treated with auxin and the control at 21 dpt. Scale bars = 0.5 cm. CK—microshoots treated with water; TM—microshoots treated with auxin.

To study the influence of auxin-induced sugarcane AR formation, microshoots treated with an IBA+NAA mixture (referred to as auxin hereafter) or water were carefully removed from the soil, and the number and length of roots were measured. The auxin treatment significantly promoted AR formation (Figure 1a,b). Thus, we hypothesized that phytohormone-responsive and other root developmental genes mediate AR formation in auxin-treated microshoots. Using RNA-seq analysis, we studied the gene expression at the basal tissues of the microshoots on the 3rd and 7th days of auxin or water treatment.

### 2.2. Transcriptome Sequencing and Assembly

To determine the transcriptional landscape in the basal tissue of sugarcane microshoots treated with auxin, twelve cDNA libraries produced from samples collected at different time periods were sequenced using the Illumina NovaSeq platform. In total, 153 Gb of high-quality sequences were obtained from the transcriptome libraries, ranging from 10.8 to 14.4 Gb per sample (Supplementary Materials Table S1). Pearson’s correlation coefficients were used to assess the reproducibility of the sequencing data. All biological replicates were correlated (R^2^ ≥ 0.90) with each other (Supplementary Materials Figure S1).

After removing low-quality regions and adaptors, we obtained a total of 512,559,594 clean reads, with Q20 values from 97.28% to 97.64%. The clean reads were then used for de novo assembly analysis by Trinity software [22]. As a result, the sequencing data were assembled into 128,094 transcripts with lengths ranging from 199 to 24,125 bases (mean length = 598 base), and 116,027 unigenes were generated (mean length = 511 bases). The total length of the unigenes was 59.4 Mb (59,370,633 bases; additional file: Supplementary Materials Table S2). The GC percentage of the assembled transcripts was 49.53%. The N50 statistic was 1571, which represents the length of at least 50% of all transcripts, including those that were 1571-bp long. The distribution of transcript and unigene lengths is shown in Supplementary Materials Figure S2. All these data indicate that the throughput was sufficiently high and that the assembly quality was adequate for further analysis. For ease of description, the samples were designated according to the time at which sampling was done following auxin treatment. Thus, the samples TM3 and TM7 represent auxin-treated sugarcane microshoots collected at 3 and 7 dpt, respectively, while CK3 and CK7 correspond to control microshoot samples.

### 2.3. Transcription Annotation

To understand their function, the assembled transcripts were annotated by mapping to the NCBI nonredundant protein (NR), Swiss-Prot, Gene Ontology (GO), Clusters of Orthologous Groups (COG)/Eukaryotic Orthologous Groups (KOG), and Kyoto Encyclopedia of Genes and Genomes (KEGG) databases.

In total, 55,373 unigenes were annotated in the databases in this study (Supplementary Materials Table S3), and most of the unigenes were annotated in the NR database. The NR mapping results showed that 15,508 (32.15%), 5144 (10.67%), 1005 (2.08%), and 972 (2.02%) of the assembled transcripts were aligned to *Sorghum bicolor*, *Zea mays*, *Setaria italica*, and *Panicum millaceum*, respectively (Supplementary Materials Figure S3).

A total of 28,705 unigenes were assigned to GO terms within the biological process, molecular function, and cellular component categories (Supplementary Materials Table S4, Supplementary Materials Figure S4). According to GO classification, most annotations were in the cellular component category, for which cell, cell part, and organelle were the top-3 GO terms. The next biggest category of annotations was in biological process, for which metabolic process, cellular process, and biological regulation were the top-3 GO terms. The third biggest group was related to molecular function, for which catalytic activity, binding, and structural molecule activity were the top-3 GO terms.

Based on the related biochemical pathways, the KEGG database provides an alternative functional annotation of genes. A KEGG pathway analysis annotated 31,595 unigenes and 363 KEGG pathways. According to the KEGG annotation, the largest group of annotations was involved in different metabolic pathways, and most unigenes were annotated in the ribosome and protein processing in the endoplasmic reticulum (Supplementary Materials Table S5).

Furthermore, we compared unigenes with the KOG classification, and 30,348 unigenes were found to be aligned to the database and distributed into 25 categories (Supplementary Materials Figure S5). Among them, the top 3 classes were general function prediction only, post-translational modification protein turnover and translation, and ribosomal structure and biogenesis. These results may provide a valuable resource for further studies on auxin-responsive genes associated with AR formation.

### 2.4. Differential Expression and Gene Functional Classification

Genes with more than a two-fold change and a false discovery rate (FDR) <0.01 were determined as differentially expressed genes (DEGs) in response to auxin treatment in the basal part of sugarcane microshoots. DEGs were identified in the pairwise comparison of TM3 compared to CK3 (referred to as CK3 vs. TM3, hereafter) and TM7 compared to CK7 (referred to as CK7 vs. TM7, hereafter), using fragments per kilobase of transcript per million (FPKM).

**Figure 2:**
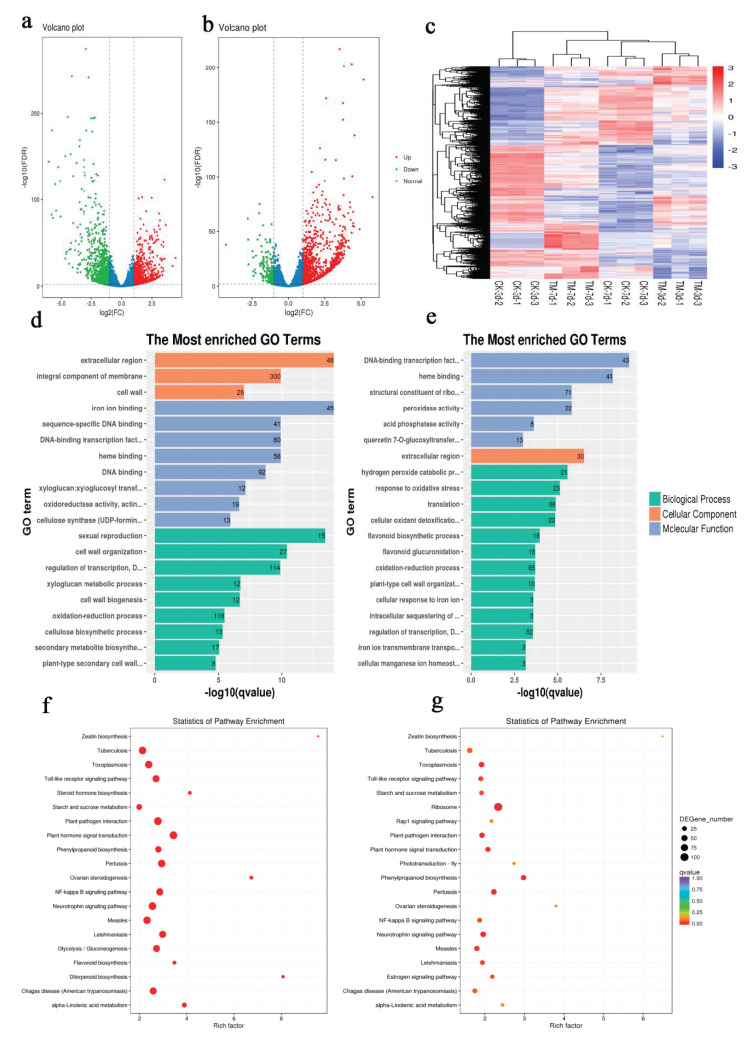
Statistics of differentially expressed genes (DEGs) identified in different stages of sugarcane microshoot tissue treated with auxin. Enriched Gene Ontology (GO) terms and KEGG enrichment in sugarcane microshoots in response to auxin. (**a**) Volcano plot showing the upregulated and downregulated genes in TM3 compared to CK3. Each point in the volcano plot represents a gene. The green points represent downregulated genes, the red points represent upregulated genes, and the black points represent unchanged genes. (**b**) Volcano plot showing the upregulated and downregulated genes in TM3 compared to CK3. The green points represent downregulated genes, the red points represent upregulated genes, and the black points represent unchanged genes. (**c**) Hierarchical cluster analysis. Different columns in the figure represent different samples, and different rows represent different genes. The colors from blue to yellow indicate increasing gene expression levels. (**d**,**e**) Significantly enriched GO terms (*p* < 0.05) in CK3 vs. TM3 and in CK7 vs. TM7. GO terms belonging to biological process, cellular component, and molecular function are shown in green, orange, and blue, respectively. (**f**,**g**) Top 20 pathways of DEGs in CK3 vs. TM3 and in CK7 vs. TM7.

In total, there were 1737 DEGs in CK3 vs. TM3, with 1001 genes downregulated and 736 genes upregulated (Figure 2a, Supplementary Materials Table S6). In contrast, out of 1268 DEGs in the CK7 vs. TM7 comparison, only 201 genes were downregulated and 1067 were upregulated (Figure 2b, Supplementary Materials Table S7). As shown in Figure 2c, the hierarchical cluster (H-cluster) analysis of all DEGs was performed to show the expression pattern of DEGs in different samples.

To gain insights into the function of DEGs, a GO term enrichment analysis of DEGs was performed (Supplementary Materials Figures S6 and S7). Most DEGs in CK3 vs. TM3 were assigned to metabolic process, cellular process, biological regulation, and response to stimulus in the category of biological process (Supplementary Materials Table S8), cell part, cell, and cell membrane in the category of cellular component (Supplementary Materials Table S9), and binding, catalytic activity, and transcription regulator activity in the category of molecular function (Supplementary Materials Table S10). The most highly enriched GO terms (top 20) among these DEGs are shown in Figure 2d and Supplementary Materials Table S11. Among them, DNA-binding transcription factor (GO:0003700), xyloglucan:xyloglucosyl transferase (GO:0016762), and cellulose synthase (GO:0016760) in the category of molecular function, and cell wall organization (GO:0071555) and cell wall biogenesis (GO:0042546) in the category of biological process might have important roles in AR formation. In the comparison between CK7 and TM7 (Supplementary Materials Table S12), as shown in Figure S7, most DEGs were assigned to metabolic process, cellular process, biological regulation, response to stimulus in the category of biological process (Supplementary Materials Table S13), cell part, cell, and membrane in the category of cellular component (Supplementary Materials Table S14), and binding, catalytic activity, and structural molecule activity in the category of molecular function (Supplementary Materials Table S15).

The most highly enriched GO terms (top 20) among these DEGs are shown in Figure 2e, which include DNA-binding transcription factor (GO:0003700), peroxidase activity (GO:0004601), response to oxidative stress (GO:0006979), flavonoid biosynthetic process (GO:0009813), and plant-type cell wall organization (GO:0009664). The GO enrichment analysis indicated that cell wall modification and transcription factor activity might be associated with AR formation induced by auxin.

To further investigate the function of DEGs, KEGG enrichment analysis was performed, and the DEGs of CK3 vs. TM3 and CK7 vs. TM7 were assigned to 210 and 209 KEGG pathways, respectively. The significantly enriched pathways for these two groups are shown in Figure 2f,g. In the CK3 and TM3 groups (Supplementary Materials Table S16), 21 KEGG pathways were found to be enriched (*q*-value < 5%), including those associated with plant hormone signal transduction (ko04075), phenylpropanoid biosynthesis (ko00940), flavonoid biosynthesis (ko00941), alpha-linolenic acid metabolism (ko00592), diterpenoid biosynthesis (ko00904), and zeatin biosynthesis (ko00908). In the CK7 and TM7 groups (Supplementary Materials Table S17), the DEGs were assigned to 209 KEGG pathways; among the top 20 pathway entries, phenylpropanoid biosynthesis (ko00940), plant hormone signal transduction (ko04075), linolenic acid metabolism (ko00592), and zeatin biosynthesis (ko00908) were included. The KEGG enrichment analysis showed that plant hormone signal transduction, phenylpropanoid biosynthesis, and flavonoid biosynthesis were similarly enriched in the two groups. Our results are consistent with previous transcriptome analyses in tea [23] and mung bean [24], in which DEGs enriched in KEGG pathways included plant hormone signal transduction, phenylpropanoid biosynthesis, and flavonoid biosynthesis. These pathways might have a key role in AR induction.

Taken together, the above results indicate that the treatment of microshoots with auxin enhances AR formation, which is closely associated with plant hormone signal transduction, flavonoid and phenylpropanoid biosyntheses, cell wall organization, and transcription factor activity.

### 2.5. Sugarcane Genes Responding to Auxin Treatment

We next analyzed the DEGs to identify genes responding to auxin treatment, especially those involved in hormone production and signaling, flavonoid and phenylpropanoid biosynthesis, cell cycle, cell wall modification, and transcription factors.

#### 2.5.1. Plant Hormone-Related Genes

Numerous DEGs encoding proteins related to plant hormone biosynthesis and signal transduction were found in CK3 vs. TM3 and CK7 vs. TM7 comparisons.

**Figure 3:**
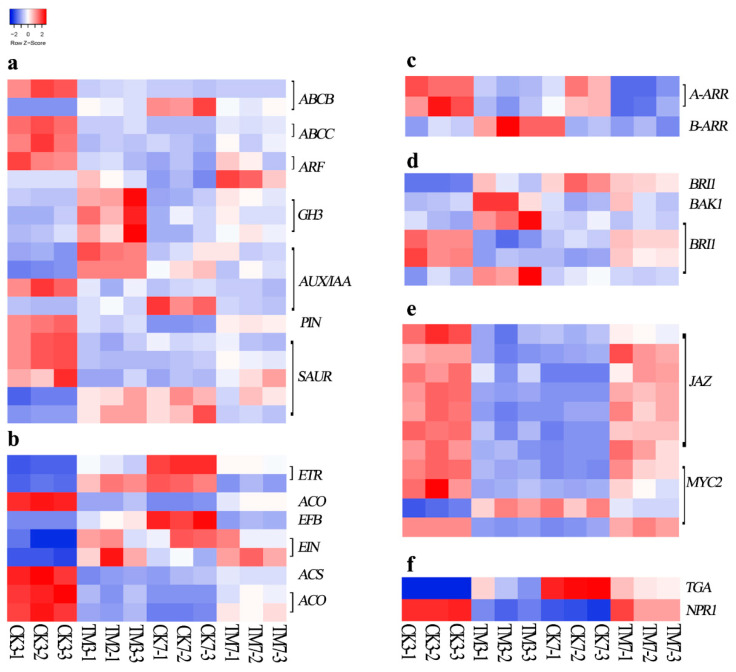
Heat map of genes involved in plant hormone transduction pathways. (**a**) Auxin, (**b**) ethylene, (**c**) cytokine, (**d**) brassinosteroid, (**e**) jasmonic acid, and (**f**) salicylic acid. The color key represents log2-transformed fold changes: red indicates high expression, and blue represents low expression. Heat map representing transcripts per million (TPM) expression from RNA-seq. The Z-score scale is shown in the top-left corner, and it ranges from red to blue.

In the auxin-signaling pathway (Figure 3a, Supplementary Materials Table S18), 19 genes were differentially expressed in the CK3 vs. TM3 comparison; however, only 4 genes were differentially expressed in CK7 vs. TM7. Three genes encoding the AUX/IAA protein were upregulated, and one gene encoding the AUX/IAA protein was downregulated in CK3 vs. TM3, whereas in the CK7 vs. TM7 comparison, only one gene encoding an AUX/IAA family protein was downregulated. An auxin response factor (ARF) was downregulated in CK3 vs. TM3; however, a gene encoding ARF was upregulated in CK7 vs. TM7. In CK3 vs. TM3, most *SAUR* genes were upregulated. However, a lot of genes encoding SAUR proteins were downregulated in the CK7 vs. TM7 group. In the present study, *ARF*, *AUX/IAA*, and *SAUR* were differentially expressed in both CK3 vs. TM3 and CK7 vs. TM7 comparisons. In addition, DEGs associated with auxin transporters were identified and found only in the CK3 and TM3 groups; most of them were downregulated in auxin-treated microshoots, such as the two genes encoding the subfamily C (CFTR/MRP) ATP-binding cassette, the one gene encoding the subfamily B (MDR/TAP) ATP-binding cassette, and another gene encoding PIN-LIKES 7 protein.

Numerous DEGs related to ethylene (ET) biosynthesis and the ET signaling pathway were found in our analysis (Figure 3b, Supplementary Materials Table S18). Three genes encoding 1-aminocyclopropane-1-carboxylate oxidase (ACO) and another one encoding 1-aminocyclopropane-1-carboxylate synthase (ACS) were differentially expressed in CK3 vs. TM3 and CK7 vs. TM7. The DEGs in the ET signaling pathway were all upregulated in CK3 vs. TM3, including two genes encoding the ethylene receptor (ETR), one gene each encoding ethylene-insensitive protein 2 (EIN2) and ethylene-insensitive protein 3 (EIN3), and another gene encoding EIN3-binding F-box protein (EBF). However, a gene encoding ETR and another one encoding EBF were downregulated in CK7 vs. TM7.

As shown in Figure 3c and Supplementary Materials Table S18, DEGs encoding ARABIDOPSIS RESPONSE REGULATOR (ARR) proteins involved in the cytokinin-signaling pathway were identified in CK3 vs. TM3 and CK7 vs. TM7. Two genes encoding A-ARR (TRINITY_DN2975_c0_g1, TRINITY_DN2975_c0_g2) were both downregulated in CK3 vs. TM3 and CK7 vs. TM7. In addition, a gene encoding B-ARR (TRINITY_DN921_c2_g2) was found to be upregulated only in the CK3 vs. TM3 comparison.

As shown in Figure 3d and Supplementary Materials Table S18, one gene (TRINITY_DN11822_c1_g2) encoding BAK1 and most genes (TRINITY_DN10833_c0_g1, TRINITY_DN1852_c4_g1, TRINITY_DN947_c1_g1) encoding BRI1 were upregulated in the CK3 and TM3 groups; however, all *B-ARR*, *BAK1,* and *BRI1* were not differentially expressed in CK7 vs. TM7.

Several DEGs (Figure 3e, Supplementary Materials Table S18) in the jasmonic acid signaling pathway were found to be induced by auxin in our study, including genes in the JAZ family and MYC. In the CK3 and TM3 groups, most DEGs, which are involved in jasmonic acid signaling, encoding JAZ proteins and MYC2, were downregulated, whereas they were upregulated in the CK7 and TM7 groups.

Salicylic acid (SA) production, which usually occurs as a response to various stresses, has also been shown to regulate plant development. PATHOGENESIS-RELATED GENE 1(NPR1) and *TGA* are important components in the SA-signaling pathway. As shown in Figure 3f and Supplementary Materials Table S18, *NPR1* showed downregulation at both timepoints, while *TGA* was upregulated at 3 dpt.

#### 2.5.2. Flavonoid and Phenylpropanoid Biosynthesis-Related Genes

**Figure 4:**
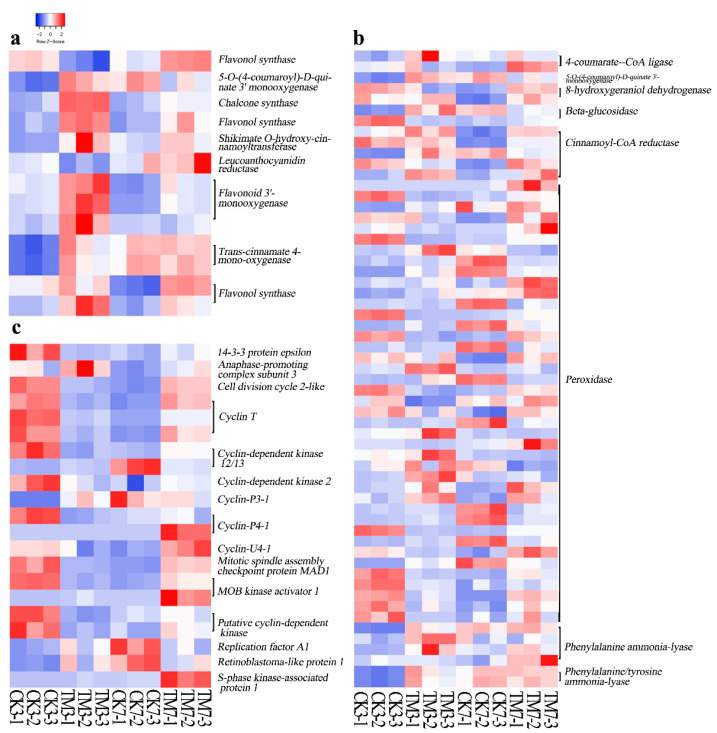
Heat map of DEGs related to flavonoid biosynthesis, phenylpropanoid biosynthesis, and the cell cycle. (**a**) Flavonoid biosynthesis, (**b**) phenylpropanoid biosynthesis, (**c**) the cell cycle. The color key represents log2-transformed fold changes: red indicates high expression, while blue represents low expression. Heat map representing transcripts per million (TPM) expression from RNA-seq. The Z-score scale is shown in the top-left corner, and it ranges from red to blue.

Three days after sugarcane microshoots were exposed to auxin, 11 DEGs involved in the flavonoid biosynthesis pathway were detected, and most of them were upregulated (Figure 4a, Supplementary Materials Table S18). This includes genes encoding trans-cinnamate 4-monooxygenase, chalcone synthase (CHS), shikimate O-hydroxycinnamoyltransferase, flavonoid 3′-monooxygenase, flavonol synthase (FLS), and 5-O-(4-coumaroyl)-D-quinate 3′-monooxygenase. In contrast, at 7 dpt, only three DEGs, flavonoid 3′-monooxygenase, FLS, and CHS, were found to be upregulated. The gene (TRINITY_DN19078_c0_g1) encoding CHS showed more than a 2-fold increase in both CK3 vs. TM3 and CK7 vs. TM7 comparisons.

In our study, 34 and 32 DEGs involved in phenylpropanoid biosynthesis were identified in CK3 vs. TM3 and CK7 vs. TM7, respectively (Figure 4b, Supplementary Materials Table S18). Among these DEGs, many encoding peroxidases showed remarkable differential expression patterns in both groups. Other DEGs, such as cinnamoyl-CoA reductase, phenylalanine/tyrosine ammonia-lyase, and trans-cinnamate 4-monooxygenase identified in the CK3 and TM3 comparison, were mostly upregulated under the auxin treatment. Moreover, a similar trend was observed for DEGs encoding 4-coumarate-CoA ligase, 5-O-(4-coumaroyl)-D-quinate 3′-monooxygenase, 8-hydroxygeraniol dehydrogenase, beta-glucosidase, cinnamoyl-CoA reductase, and sinapate 1-glucosyltransferase in the CK7 vs. TM7 comparison in response to the auxin treatment. Most genes encoding phenylalanine ammonia-lyase (PAL) were downregulated in CK3 vs. TM3, whereas the majority of them were upregulated in CK7 vs. TM7. The altered expression of these genes involved in phenylpropanoid biosynthesis might cause alterations in the production of lignin and many functional compounds, including flavonoids involved in various processes associated with cell division and organogenesis.

#### 2.5.3. Cell Cycle-Related Genes

In the present study, most DEGs associated with the cell cycle were downregulated in CK3 vs. TM3, whereas most of these DEGs were upregulated in CK7 vs. TM7 (Figure 4c, Supplementary Materials Table S18). Genes encoding cyclin T were all downregulated in CK3 vs. TM3 and upregulated in CK7 vs. TM7. One gene encoding retinoblastoma-like protein 1 was found to upregulated in CK3 vs. TM3. A gene (TRINITY_DN39410_c0_g1) encoding cyclin-P4-1 was downregulated in CK3 vs. TM3, and a gene (TRINITY_DN133286_c0_g1) encoding cyclin-U4-1 was downregulated in CK7 vs. TM7. Most genes (TRINITY_DN17303_c0_g1, TRINITY_DN355_c1_g1, TRINITY_DN23652_c0_g2, TRINITY_DN2462_c2_g1) encoding cyclin-dependent kinase (CDK) were downregulated in CK3 vs. TM3, and another gene (TRINITY_DN3656_c0_g1) encoding CDK was downregulated in CK7 vs. TM7.

#### 2.5.4. Cell Wall Modification-Related Genes

**Figure 5:**
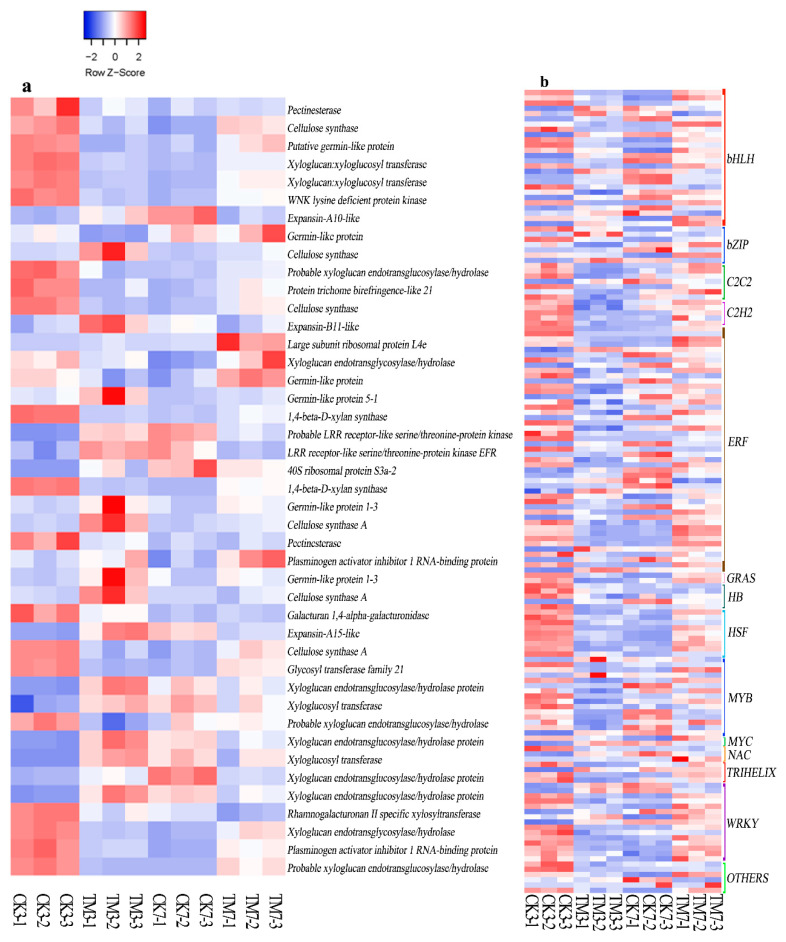
Heat map of DEGs related to cell wall modification, and transcription factors. (**a**) Cell wall modification-related genes, (**b**) transcription factors. The color key represents log2-transformed fold changes: red indicates high expression, while blue represents low expression. Heat map representing transcripts per million (TPM) expression from RNA-seq. The Z-score scale is shown in the top-left corner, and it ranges from red to blue.

Several genes involved in cell wall modification were also found to be differentially expressed after auxin application (Figure 5a, Supplementary Materials Table S18). Two genes (TRINITY_DN11826_c0_g1, TRINITY_DN1254_c0_g1) encoding xyloglucan:xyloglucosyl transferase, two genes (TRINITY_DN6522_c0_g1, TRINITY_DN9183_c0_g2) encoding xyloglucan endotransglycosylase/hydrolase, one gene (TRINITY_DN10772_c0_g1) encoding cellulose synthase, and another one encoding putative germin-like protein (TRINITY_DN11032_c1_g1) were downregulated in CK3 vs. TM3 and upregulated in CK 7 vs. TM7. One gene each, (TRINITY_DN6198_c0_g2) encoding xyloglucan endotransglucosylase/hydrolase protein, (TRINITY_DN16511_c0_g1) expansin-A10-like, and (TRINITY_DN54970_c0_g1) expansin-A15-like, were upregulated in CK3 vs. TM3 and downregulated in CK7 vs. TM7 comparisons.

#### 2.5.5. Transcription Factors

Numerous plant TFs are involved in plant development, including AR formation. We analyzed the sequence data to identify TFs associated with AR formation (Figure 5b, Supplementary Materials Table S18). Our results showed that the expression of members of the bHLH, bZIP, C2C2, C2H2, ET-responsive TF (ERF), GRAS, HB, HSF, MYB, NAC, TRINELIX, Homodox, and WRKY TF families were significantly altered after the auxin treatment. A total of 131 TFs were differentially expressed in CK3 vs. TM3, with 89 TFs downregulated and 42 upregulated; this included 19 bHLH, 5 bZIP, 40 ERF, and 11 WRKY. In the CK7 vs. TM7 comparison, 22 ERFs, 14 bHLHs, and 8 WRKYs were differentially expressed; this included 14 bHLHs, 4 bZIPs, 23 ERFs, 2 HSFs, and 18 WRKYs. Most TFs were downregulated in CK3 vs. TM3, but an opposite trend was observed in CK7 vs. TM7. Notably, we found that the ERF family had the highest number of differentially expressed TFs in both CK3 vs. TM3 and CK7 vs. TM7. In addition, there were also many bHLH and WRKY genes in these two experimental groups. Auxin treatment downregulated the gene (TRINITY_DN11556_c0_g1) encoding scarecrow-like protein 4 in CK3 vs. TM3 but upregulated it in CK7 vs. TM7. In addition, a DEG (TRINITY_DN18674_c0_g2) encoding scarecrow-like protein 6 was found upregulated only in CK3 vs. TM3.

### 2.6. Verification of DEGs Using Quantitative Reverse Transcription–Polymerase Chain Reaction Analysis

**Figure 6:**
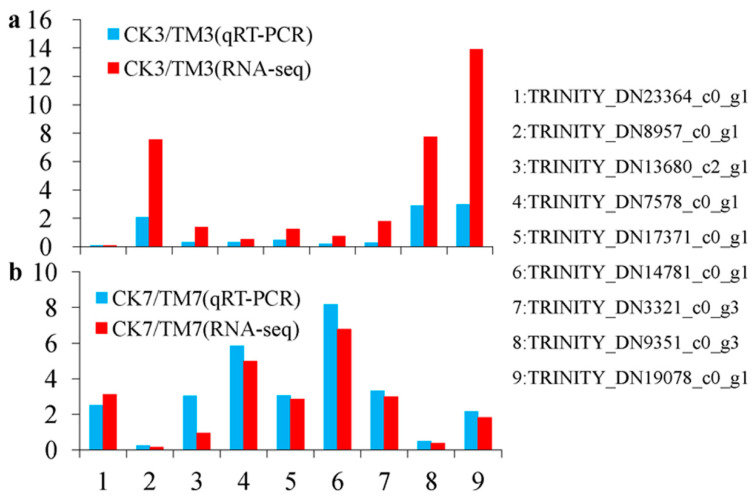
DEGs confirmed by qRT–PCR using the same samples as in the RNA-seq. (**a**) Results from CK3 vs. TM3 and (**b**) CK7 vs. TM7. The blue column represents the qRT–PCR results and the red column represents the RNA-seq results. The *y*-axis represents the fold change of the relative expression levels of DEGs.

Quantitative reverse transcription–polymerase chain reaction analysis (qRT–PCR) was performed with nine genes (TRINITY_DN23364_c0_g1, SAUR family protein; TRINITY_DN8957_c0_g1, auxin-responsive protein IAA; TRINITY_DN13680_c2_g1, EREBP-like factor; TRINITY_DN7578_c0_g1, ethylene-responsive transcription factor ERF039; TRINITY_DN17371_c0_g1, P450 87A3 isoform X1; TRINITY_DN14781_c0_g1, jasmonate O-methyltransferase; TRINITY_DN3321_c0_g3, cis-zeatin O-glucosyltransferase; TRINITY_DN9351_c0_g3, peroxidase; TRINITY_DN19078_c0_g1, chalcone synthase) selected from the sequence database to validate the results of the Illumina RNA-seq. These genes were selected because they are thought to be associated with AR, and we also wanted to include up- and downregulated genes for a quantitative study. SAUR family protein, auxin-responsive protein IAA, is related to the “plant hormone signal transduction pathway”, namely, the KEGG pathway. EREBP-like factor and ethylene-responsive transcription factor ERF039 are TFs implicated in rooting. Enzymes peroxidase is associated with phenylpropanoid biosynthesis, and chalcone synthase is associated with flavonoid biosynthesis, which are both linked to rooting. Additionally, P450 87A3 isoform X1, jasmonate O-methyltransferase, and cis-zeatin O-glucosyltransferase were chosen from the genes as they have annotations against the database. The relative mRNA expression of unigenes assessed by qRT–PCR (Figure 6a,b) was very similar to the levels shown in the RNA-seq analysis, suggesting the reproducibility and accuracy of the RNA-seq results.

## 3. Discussion

The developmental transition of shoot cells to root cells is one of the least studied developmental processes in plants. This fundamental shift, similar to the phase transition of vegetative meristem to reproductive meristem during flowering, could involve endogenous cues that prime the target shoot cells to initiate distinct molecular, physiological, structural, and developmental phase transitions towards root primordium. While considerable physiological and structural aspects of this developmental transition are known [25,26,27], genes and gene networks or the mechanism(s) controlling this process in graminaceous plants remain largely unknown, and nothing is understood in sugarcane. Previous studies on *Arabidopsis*, cucumber, and tea have associated certain genes and TFs involved in plant hormone signal transduction and secondary metabolism with AR formation [20,27]. Transcriptome analysis has been used to understand and identify genes and pathways involved in cellulose and lignin biosyntheses [28], ripening [29], and leaf abscission [30] in sugarcane. However, it was never applied to identify genes related to AR formation in sugarcane. In this study, many genes induced by auxin, likely to be involved in or at least associated with AR formation, were identified.

**Figure 7:**
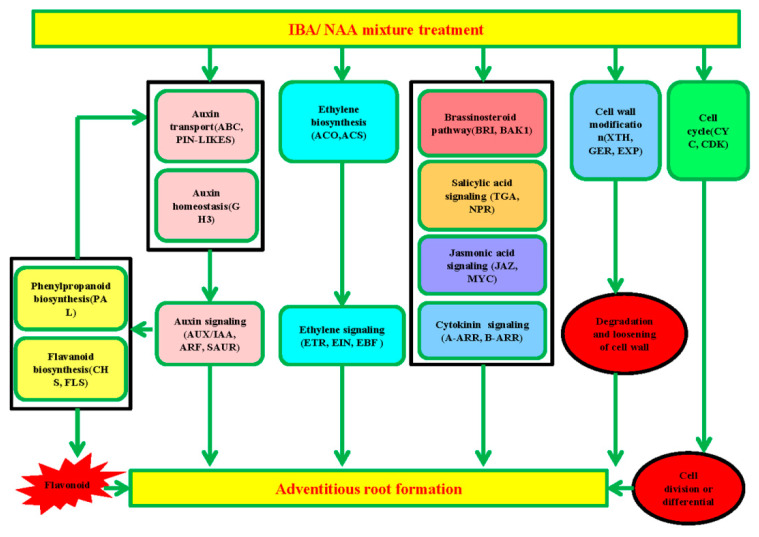
A possible functional network of the AR induction pathway in sugarcane. After supplementation with exogenous IBA and NAA mixture, proteins related to auxin homeostasis and transport facilitated auxin accumulation in sugarcane, which promoted auxin signaling. Enhanced ethylene biosynthesis accompanied changed ethylene signaling. Meanwhile, the cytokinin, brassinosteroid, jasmonic acid, and salicylic acid signals were affected. Auxin signaling plays a positive role in stimulating phenylpropanoid biosynthesis and flavonoid biosynthesis. Flavonoids, which are produced via phenylpropanoid biosynthesis and flavonoid biosynthesis, can affect auxin transportation. The accumulation of flavonoids induced by auxin can promote AR formation. Genes related to cell wall modification are also associated with AR formation through the degradation and loosening of the cell wall, which might be the limiting factor for rooting ability. In addition, the cell cycle plays a key role in cell division and differentiation, and this process is also critical for AR formation. However, the gene that determines cell fate transition remains elusive.

The different rooting abilities of sugarcane microshoots exposed to auxin and water are likely to be due to the differential expression of many key genes in some important metabolic and developmental pathways. As a result, the number and length of ARs were increased by the auxin treatment (Figure 1a,b). Several genes encoding key proteins related to plant hormone signaling, flavonoid biosynthesis, phenylpropanoid biosynthesis, the cell cycle, cell wall modification, and TFs were differentially expressed during AR induction in sugarcane. Detailed information on these genes is shown in Supplementary Materials Table S18. Considering these cellular processes associated with DEGs, the developmental transition of shoot cells to root meristem involves multiple hormone biosynthesis and signaling, cell proliferation, and cellular structural differentiation, and a putative model of AR development in sugarcane microshoots is proposed (Figure 7). These aspects are further explored below.

### 3.1. Genes Related to Plant Hormone Signaling

Phytohormone-related genes, particularly those associated with auxin, ethylene, cytokinin, jasmonic acid, and salicylic acid, play important roles in regulating AR formation [20].

Many DEGs related to auxin signaling, such as *AUX/IAA*, *ARF*, *SAUR*, *GH3*, and *PIN-LIKES*, were identified in our study. The *ARF* gene family, together with AUX/IAA proteins, regulates auxin-mediated transcriptional activation/repression [31]. *ARF6* and *ARF8* are positive regulators in *Arabidopsis* AR formation induced by auxin [19]. In contrast, in sugarcane, one *ARF* gene (TRINITY_DN10673_c0_g1) was downregulated on the third day, and another one, *ARF* (TRINITY_DN3251_c0_g2), was upregulated on the seventh day after auxin application. However, these different *ARF* and *AUX/IAA* genes have various redundancy roles in the regulation of the plant’s biological and physiologic processes [32]. The results showed that during auxin-induced AR formation, most of these DEGs showed opposite expression patterns in the early and late stages of root development, probably because these DEGs may play different developmental regulatory roles at different stages of AR formation. In addition, GH3 proteins, which play a crucial role in conjugating IAA to amino acids, are critical in maintaining auxin homeostasis [33]. In the moss *Physcomitrella patens*, the knockout of *GH3* genes increased sensitivity to auxin, causing growth inhibition [34]. All *GH3* genes (TRINITY_DN2401_c0_g2, TRINITY_DN638_c2_g1, TRINITY_DN15418_c0_g1) were upregulated in auxin-treated sugarcane microshoot basal tissue at 3 dpt. Regulation of the subfamily of ATP-binding cassette (ABC) transporters, which have been demonstrated to regulate the polar auxin transporter (PAT), was associated with AR formation [18]. Three (TRINITY_DN1885_c0_g2, TRINITY_DN483_c0_g1, and TRINITY_DN93602_c0_g1) of the four genes encoding ABC transporters were downregulated after auxin treatment at 3 dpt. PIN-LIKES is a family of putative auxin transporters [12], and decreasing its activity resulted in more abundant and longer lateral roots, suggesting its specific root development functions [35]. In our study, the *PIN-LIKES* showed lower expression in auxin-treated microshoots at 3 dpt. The downregulation of PIN-LIKES and ABC transporter in our results suggests that auxin transporters are likely to be important components of auxin-induced AR formation. The genes were differentially expressed in only the first stage, suggesting that the mechanism associated with auxin homeostasis was involved in the early root induction stage and their activity changes in a time-dependent manner. A previous study demonstrated that expression of *AUX/IAA*, *SAUR*, and *GH3* family proteins was affected by auxin [36]. During AR formation in *Taxus media* stem cuttings, most auxin-related genes, including *AUX/IAA, ARF*, *GH3,* and *SAUR,* were upregulated after auxin treatment [37]. Our results are consistent with the results of these studies, suggesting that the genes related to auxin response, homeostasis, and transport could be involved in auxin-induced AR formation in sugarcane.

Ethylene functions as a positive regulator of AR formation in many plant species [38]. Ethylene biosynthesis and perception are essential for AR formation [39]. ACO and ACS are key enzymes in the ET biosynthesis pathway [40]. Transcript levels of ACS and ACO in plants were enhanced after auxin application [41]. In our study, all genes involved in ET biosynthesis were downregulated at 3 dpt, whereas these genes were upregulated at 7 dpt. EIN3/EIL1 integrates ET and JA signaling during plant root hair formation, which physically affects the removal of JA-Zim-domain (JAZ) proteins [42]. ET signal transduction-related genes showed contrasting expression patterns at the two stages (3 and 7 dpt) of AR formation, induced by the auxin studied here. Therefore, our results show that the ET-dependent signal cascade might play a developmental-stage-dependent role in sugarcane AR formation.

ARR is an important regulator in the cytokinin signaling pathway and it has two main types, type A and type B. Type B ARRs positively regulate CTK signaling, whereas type A ARRs negatively regulate the CTK response and are transcriptionally regulated by type B ARRs [43]. Enhancing the expression of the type B CTK response regulator reduced the number of ARs [44]. Reducing the expression level of type B CTK receptors (ARR1, ARR10, ARR12) induced AR formation [45]. All the genes encoding type A ARRs were downregulated at 3 and 7 dpt; however, the type B ARR gene showed upregulation at 3 dpt. These results indicate that differential expression of type A and B ARRs may be involved in sugarcane AR formation induced by auxin.

In our study, the transcript abundance of BAK1 and BRI1 genes in the BR signaling pathway showed significant changes only on the third day after auxin application, with most of them being upregulated (Figure 3d). BRs are perceived by the cell surface receptor kinase BRI1 in plants [46]. The size of the root meristem is controlled by BRI1 activity in the epidermis [47].

Jasmonic acid (JA) is also an important regulator of AR formation. Depending on the species, JA has been shown to be a positive or a negative regulator of AR formation [48,49]. JAZ and MYC genes, which respond to JA/JA–Ile, are negative and positive regulators of the expression of JA biosynthesis genes, respectively [50]. *ETR*, *CTR1*, *EIN2*, *EIN3,* and *ERFP1/2* in the ET signaling pathway, *BAK1* and *BRI1* in the BR pathway, and *JAZ* and *MYC2* were differentially expressed during AR formation [42]. Taking these results together, many genes involved in auxin, ET, BR, JA, CTK, and SA signaling were found to be associated with, and thus possibly involved in, sugarcane AR formation.

### 3.2. Genes Related to Flavonoid and Phenylpropanoid Biosyntheses

It has been widely reported that secondary metabolism pathways, including those of flavonoids, terpenoids, and phenylpropanoids, are involved in auxin-induced AR formation [23].

Our results revealed that a large number of DEGs are involved in phenylpropanoid and flavonoid biosynthesis pathways. For example, chalcone synthase, flavonoid 3′-monooxygenase, and flavonol synthase genes were upregulated in auxin-treated microshoots at both 3 and 7 dpt (Figure 5a). An increase in the accumulation of endogenous flavonoids is known to contribute to AR formation [51]. In *Arabidopsis thaliana*, auxin treatment caused an increase in CHS and FLS transcript abundance and kaempferol and quercetin content [52]. Flavonoids such as quercetin, kaempferol, apigenin, and other aglycone molecules synthesized in the flavonoid biosynthetic pathway inhibit polar auxin transport and enhance localized auxin accumulation in plants [53]. Therefore, considering our results, flavonoid biosynthesis genes are likely to be involved in auxin-induced sugarcane microshoot AR formation.

Many derivatives from the phenylpropanoid pathway are important regulators in cell division and differentiation [54]. Several genes involved in the phenylpropanoid pathway were differentially expressed at 3 and 7 dpt. For example, genes encoding phenylalanine ammonia-lyase in auxin-treated stems were either downregulated (TRINITY_DN26709_c0_g2, TRINITY_DN35873_c0_g3, TRINITY_DN422_c1_g1) or upregulated (TRINITY_DN2703_c0_g2, TRINITY_DN35873_c0_g3), depending on the developmental stage. In *Arabidopsis*, changes in the early steps of the phenylpropanoid pathway play an important role in plant development. PAL converts l-phenylalanine into trans-cinnamic acid, which promotes growth in *Arabidopsis* at low concentrations [55]; a similar activity may occur in auxin-treated microshoots as well.

### 3.3. Cell Cycle

Cyclin-dependent kinases and cyclins have been shown to function as key regulators in auxin-mediated regulation of the cell cycle [56]. Our study demonstrated that the expression of most genes related to the cell cycle was decreased in CK3 vs. TM3 and increased in CKT vs. TM7. *Cyclin*s and *CDK*s showed downregulation at 3 dpt, whereas they increased their expression at 7 dpt, suggesting a change in cell-cycle dynamics during AR formation in auxin-treated sugarcane. The RETINOBLASTOMA-RELATED (RBR) gene, which is a key cell-cycle regulator, has an important role in the control of cell division and differentiation [17]. *RBR* (TRINITY_DN58083_c0_g1) was found to be downregulated at 3 dpt, indicating a role for *RBR* for the induction of the cell during auxin-induced AR formation in microshoots. Localized reduction of RETINOBLASTOMA-RELATED (RBR) gene expression in *Arabidopsis* roots increases the number of stem cells; however, iRBR overexpression dissipates stem cells prior to arresting other mitotic cells [57]. The reduction of RBR levels leads to asymmetric cell divisions in the quiescence center (QC) cells of the stem cell niche, thus regenerating short-term stem cells [58]. Our study shows that auxin might promote cell division and accelerate root regeneration in sugarcane basal stem via the reduction of *RBR* transcript abundance.

### 3.4. Cell Wall Modification

During AR formation, the cell wall undergoes changes, and rooting is promoted by enzymes that degrade cell walls, such as cellulase and pectinase [30], suggesting that cell wall modification is essential for AR formation. It was reported that auxin regulates the expression of genes encoding cell wall enzymes that modulate the properties of the cell wall, including subtilisin-like protease 7, pectate lyase 23, polygalacturonase 24, xyloglucan:xyloglucosyl transferase 25, expansin 26, and glycosyl hydrolase 27 [59]. Cellulose synthase showed remarkable expression at 3 dpt, and it continued at 7 dpt as well. Auxin stimulates cell elongation by increasing wall extensibility and plays a role in promoting cell expansion by modulating cell wall properties [60]. XYLOGLUCAN ENDOTRANSGLUCOSYLASE/HYDROLASEs (XTHs) and expansins were shown to be involved in loosening the plant cell wall [61,62,63]. Genes encoding expansins (TRINITY_DN16511_c0_g1, TRINITY_DN54970_c0_g1, TRINITY_DN19060_c0_g1) were regulated at 3 dpt but another one encoding expansin (TRINITY_DN16511_c0_g1, TRINITY_DN54970_c0_g1) was downregulated at 7 dpt. Similarly, genes encoding germin-like protein (TRINITY_DN23882_c0_g1, TRINITY_DN34757_c0_g1, TRINITY_DN48688_c0_g1, TRINITY_DN25484_c0_g1), which contributes to the oxidative scission of polysaccharides, are likely to be involved in AR induction [64]. Most genes showed higher expression patterns in auxin-treated microshoots at 7 dpt. Our data collectively suggest that genes associated with cell wall modification could be a critical component of AR formation in sugarcane microshoots.

### 3.5. Transcription Factors

Transcription factors have important roles in plant hormone biosynthesis and signaling, cell growth and differentiation, and photomorphogenesis [65]. There were a large number of differentially expressed TF genes after the microshoots were treated with auxin, and among them, the ERF family showed the most pronounced activity, indicating a major role in auxin-induced adventitious rooting in sugarcane.

In addition, many bHLH and WRKY genes also showed strong differential expression during AR formation in microshoots. In poplar, the AP2/ERF, MYB, NAC, WRKY, and bHLH families were significantly expressed during the early stages of AR formation, of which the MYB and AP2/ERF families were the most highly modulated [66]. Most MYBs were significantly downregulated in auxin-treated microshoots. MYB TFs are involved in the regulation of plant metabolism, development, and responses to biotic and abiotic stresses [67]. bHLH is another important TF that might be associated with AR formation. In *Arabidopsis*, transcript levels of bHLH are associated with root length [68]. In sugarcane microshoots, bHLH was downregulated at 3 dpt and upregulated at 7 dpt. WRKY genes, involved in plant root development, root hair development, and flavonoid synthesis [69,70], are thought to regulate lateral root formation by modulating auxin transport [71]. A large number of WRKY genes showed differential expression in our study, with most of them downregulated at 3 dpt and then upregulated at 7 dpt in auxin-treated microshoots. An increasing number of studies have shown that several TF families, such as ERF, LBD, and GRAS, which include scarecrow, are involved in AR formation. In our study, auxin treatment downregulated the gene (TRINITY_DN11556_c0_g1) encoding scarecrow-like protein 4 in CK3 vs. TM3 but upregulated it in CK7 vs. TM7. However, scarecrow-like protein 6 (TRINITY_DN18674_c0_g2) was upregulated in CK3 vs. TM3 but not differentially expressed at 7 dpt. Auxin-responsive transcription factor SCR has been shown to play roles in the control of cell division, leading to root primordia differentiation and linking auxin-signaling with cell specification and patterning [17,18]. Additionally, *scr* mutant’s growth rate and meristem size were found to be reduced compared to wild-type plants. Root growth and stem cell activity were only maintained when SCR expression was restored in QC cells [72]. In wild-type plants, increased SCR expression started in the founder cells of ARs and continued to be present in primordia and elongating ARs [18]. The profound change of scarecrow-like protein genes at 3 and 7 dpt after auxin application indicates an important role for *SCR* in AR formation induce by auxin.

Plant development under normal growth conditions requires signaling pathways and transcription factor networks to regulate the activity of meristems and their constituent stem cell niches. The pair RBR–SCR has been described extensively in root development. The *RBR* gene is considered a key cell-cycle regulator downstream of the *SCR* gene [17]. In *Arabidopsis*, JA regulates organizer cell activity in the root stem cell niche through the RBR–SCR network and stress response protein ERF115. RBR–SCR operates downstream of ERF115, both in stem cell regulation and in tissue regeneration [73]. The RETINOBLASTOMA-RELATED (RBR)–SCARECROW (SCR)–SHORT ROOT (SHR) protein network regulates asymmetric cell divisions of root stem cells. RBR regulates the activity of SHR–SCR along with auxin and the CDK interactor CYCLIND6; 1 in ground tissue initial development region. In the root stem cell niche, RBR is specifically required in the ground tissue stem cell, where an accurate spatiotemporal regulation of its phosphorylation status determines its ability to bind and repress the SCARECROW transcription factor, thus regulating asymmetric cell division [74]. In this study, *RBR* and *SCR* were both found to be differentially expressed at 3dpt after auxin application, indicating that there may be an interplay of RBR and SCR activity in auxin-induced AR formation.

Our results demonstrated that TFs such as WRKY and MYB were induced by auxin in CK3 vs. TM3 and repressed in CK7 vs. TM7, suggesting that their involvement in sugarcane AR formation could be developmental-stage-specific.

## 4. Materials and Methods

### 4.1. Plant Materials and Growth Conditions

Sugarcane (*Saccharum* spp. interspecific hybrids) microshoots of cultivar 08-1589 (a promising advanced clone that has yet to be released) were used for the experiments reported here. They were generated from shoot meristem and grown and multiplied on MS medium in the tissue culture facility at the Sugarcane Research Institute (SRI), Guangxi Academy of Agricultural Sciences (GXAAS), Nanning, Guangxi, China. The experiment was carried out in a 4-m-high arch-shaped greenhouse with a plastic roof, with provision for opening on four sides, at the SRI experimental farm. After multiplication, a total of 10 microshoots (the height ranging from 5 to 10 cm and each microshoot with 2–3 leaves) was placed in each bottle with no growth medium, and then, 7 mL of the IBA and NAA mixture (composed of 50 mg/L IBA, 20 mg/L NAA, 3 g/L KH_2_PO_3_, 20 g/L sucrose, 20 g/L proline, and 2 ml/L Tween 20) was immediately sprayed in every bottle of microshoots for 8 s. The IBA and NAA levels used for rooting here was based on our preliminary experiments (data not shown) to determine the optimum level of auxins needed for maximum AR formation in sugarcane microshoots. Distilled water was used for control. After the sugarcane microshoots were treated with auxin or water, they were kept in an open bottle for 24 h in the greenhouse for acclimatization to the external environment. They were then immersed in Liangdun solution (Syngenta; fludioxonil is the active chemical) for about 10 min for sterilization; it helped to wash the excess MS medium materials from the multiplication process. These microshoots were transplanted to plastic trays containing a mixture (100 g per tray) of soil and sand at 1:1 (*v*/*v*) moistened with water containing nitrogen (0.5 mg), phosphorous (20 mg), and potassium (30 mg) per kg soil–sand mixture. The relative humidity and temperature in the greenhouse ranged from 60% to 80% and 20 to 40 °C, respectively, during the experiment. The soil was obtained from a nearby piece of uncropped land in the SRI experimental farm compound, and the sand was sourced from Sanyi Construction Co. Ltd., Nanning, Guangxi. For all treatments, the microshoots were transplanted to a plastic tray (60 cm long and 33 cm wide), each with 48 holes (8 × 6 rows). For all treatments, the experimental units for each clone were 20 plastic trays in each line, and they were replicated thrice. All plants received regular irrigation twice a day, and they were managed following SRI crop management recommendations. All plantlets that remained green were considered living and used for measurements.

For root measurements, the planted microshoots were sampled every two days after transplanting, and measyrements was conducted in sugarcane with auxin treatment and the control at 7, 9, 11, 13, 15, and 21 dpt (days post-treatment). They were very carefully rinsed with water to remove the soil attached to roots, and the adventitious root number and length were measured. For root measurement, twenty microshoots from each replicate were measured. Plants were grown for 21 dpt. For molecular studies on auxin-induced AR formation, microshoot basal tissue samples were collected on the third and seventh days post-IBA+NAA treatment.

At each time point of sampling/measurement, 5 mm basal tissue of each microshoot (both auxin-treated and control) was harvested, immediately frozen in liquid nitrogen, and then stored at −80 °C for further analyses. There were four group samples, including 3 biological controls (CK3-1, CK3-2, CK3-3; TM3-1, TM3-2, TM3-3; CK7-1, CK7-2, CK7-3; TM7-1, TM7-2, TM7–3). In total, 36 microshoots (3 microshoots for each sample × 3 biological replications × 2 treatments × 2 time points) were used for RNA-seq.

### 4.2. RNA Extraction, Library Preparation, Sequencing, and Transcriptome Assembly

Total RNA from the basal portion of sugarcane microshoots (without the adventitious root) was extracted using a Plant RNA Kit (BioTeke, Beijing, China) according to the manufacturer’s protocol. RNA integrity and purity were checked by Agilent 2100 and Nanodrop 2000 instruments (Thermo Scientific, Life Technologies, Waltham, MA, USA), respectively. The RNA concentration of each sample was quantified by agarose electrophoresis. Then, DNase I was used to remove residual genomic DNA. mRNA was purified from the total RNA by magnetic beads with oligo (dT) and then broken into small fragments. These RNA fragments were used for cDNA synthesis by a FastQuant RT Kit (Tiangen, Beijing, China). cDNA libraries were generated, and paired-end reads were obtained by an Illumina NovaSeq 6000 (Illumina Inc., San Diego, CA, USA). Clean raw reads devoid of adaptor sequences and low-quality sequences (reads with ambiguous bases (“N”), with more than 50% Q < 10 bases) were assembled using Trinity software [75]. The read counts of unigenes were calculated by the software RSEM v1.2.29, and the gene expression level was calculated using the fragments per kilobase of transcript per million (FPKM) mapped reads method.

### 4.3. Gene Functional Annotation

The assembled transcripts were regarded as the reference sequence.

Using RSEM v1.2.29 software, unigenes were mapped with the reference generated by Trinity software. The resulting unigene sequences were annotated based on the protein databases, including the NCBI nonredundant database, Swiss-Prot, Clusters of Orthologous Groups (COG), and Kyoto Encyclopedia of Genes and Genomes (KEGG) databases. Functional annotation by Gene Ontology (GO) terms (www.gene.ontology.org) was analyzed by Blast2GO software.

COG and KEGG pathway analyses were performed by sequence comparisons against the two databases using blastall and KAAS software (ftp://ftp.ncbi.nih.gov/blast/executables/release/2.2.18/). PFAM protein family alignments were performed using the HMMER 3.0 package.

### 4.4. Identification and Annotation of Differentially Expressed Genes

For the discovery of DEGs, DESeq [76] was applied with FPKM values to analyze gene expression levels, and the DEGs were screened with a fold-change ≥2 and a false discovery rate (FDR) <0.01. An FDR of 0.01 was used as the threshold of the *p*-value in multiple tests to evaluate the significance of gene expression differences. A GO enrichment analysis was performed using the GOseq R package based on the Wallenius noncentral hypergeometric distribution [77]. GO terms with corrected *p*-values < 0.05 were considered significantly enriched in DEGs. For pathway enrichment analysis, KOBAS 2.0 (https://www.biostars.org/p/200126/) was employed, and a threshold of FDR ≤ 0.05 was defined. Heatmaps and hierarchical clustering were generated with Genesis 1.8.1.

### 4.5. Validation of RNA-Seq Data by qRT–PCR

The relative expression of mRNA was quantified by quantitative reverse transcription–polymerase chain reaction (qRT–PCR) assay using sugarcane GAPDH (EF189713) mRNA as a reference, as designed by Ming H [78]. qRT–PCR was performed using Bio-Rad SYBR Green PCR Master Mix (TaKaRa, Mountain View, CA, USA), with three biological replicates for each gene and two technical repeats per experiment. Target-specific primers were designed from RNA-seq sequences using the NCBI primer designer. The relative gene expression was calculated using the 2-^ΔΔCT^ formula. The primers used in this study are listed in Supplementary Materials Table S19.

### 4.6. Statistical Analyses

The independent samples *t*-test was used to analyze the data. The analysis was conducted using SPSS 22.0 software.

## 5. Conclusions

Sugarcane microshoots of most varieties will survive and root in soil environments during photoautotrophic rooting. Our study showed that treating sugarcane microshoots with a combination of auxins IBA and NAA enhanced adventitious rooting. An analysis of the transcriptome during auxin-induced AR formation identified several genes involved in plant hormone signaling, cell wall modification, cell cycle, flavonoid and phenylpropanoid biosyntheses, and TFs that were shown to be differentially expressed in microshoots after exposure to IBA and NAA. It appears that auxin, ET, JA, and SA interact to transition shoot cells to root meristem and then to AR through cell wall modification and synthesis, cell proliferation, root meristem identity preservation, and cell growth. We constructed a putative model to illustrate the likely molecular mechanisms that are associated with auxin-mediated AR induction and growth in sugarcane (Figure 7). The current study advances our knowledge of transitioning shoot cells to roots and provides a basis for future exploration of the molecular basis of auxin-induced AR formation in graminaceous plants.

## Supplementary Materials

The following are available online at https://www.mdpi.com/2223-7747/9/8/931/s1, Figure S1: Pearson correlation of all samples, Figure S2: The distribution of transcripts and unigenes assembled from the RNA-seq data. The x-axis indicates length of the transcripts and unigenes. The y-xis indicates the number of transcripts or unigenes, Figure S3: NR annotation of unigenes obtained from RNA-seq data, Figure S4: GO annotation of unigenes obtained from RNA-seq data, Figure S5: COG classification of unigenes obtained from RNA-seq data, Figure S6: GO function annotation of differentially expressed unigene in CK3/TM3, Figure S7: GO function annotation of differentially expressed unigene in CK7/TM7, Table S1: Statistics of output sequencing in Sugarcane microshoot base, Table S2: Assemble state imformation of unigenes, Table S3: Function annotation of unigenes in different annotation database, Table S4: The number of unigenes in GO term, Table S5: KEGG pathway of unigenes, Table S6: DEGs in CK3 vs. TM3, Table S7: DEGs in CK7 vs. TM7, Table S8: Top GO term biological process in CK3 vs. TM3, Table S9: Top GO term molecular function in CK3 vs. TM3, Table S10: Top GO term cell cellular in CK3 vs. TM3, Table S11: The enriched GO term in CK3 vs. TM3, Table S12: Top GO term biological process in CK7 vs. TM7, Table S13: Top GO term molecular function in CK7 vs. TM7, Table S14: Top GO term cell cellular in CK7 vs. TM7, Table S15: The enriched GO term in CK7 vs. TM7, Table S16: The enriched KEGG pathway in CK3 vs. TM3, Table S17: The enriched KEGG pathway in CK7 vs. TM7, Table S18: The DEGs in heatmap, Table S19: Primer used for qRT-PCR.
