# Supplementary material for: Transcriptome Profiling Provides Molecular Insights into Auxin-Induced Adventitious Root Formation in Sugarcane (Saccharum spp. Interspecific Hybrids) Microshoots
Source: Plants (Basel). 2020 Jul 23;9(8):931. doi: 10.3390/plants9080931 (PMC7465322; doi:10.3390/plants9080931)
Supplement: Supplementary file 1 [file plants-09-00931-s001.zip › plants-836632-supplementary-proof/0721plants additional files/additional file imformation.pdf]

Supplementary information

Fig S1 Pearson correlations of all samples.

Fig S2 Distribution of transcripts and unigenes assembled from the RNA-seq data. The x-axis indicates the length of the transcripts and unigenes. The y-axis indicates the number of transcripts or unigenes.

Fig S3 NR annotation of unigenes obtained from RNA-seq data.

Fig S4 GO annotation of unigenes obtained from RNA-seq data.

Fig S5 COG classification of unigenes obtained from RNA-seq data.

Fig S6 GO function annotation of differentially expressed unigenes.

Fig S7 GO annotation of unigenes in CK3 vs. TM3.

Fig S8 GO annotation of unigenes in CK3 vs. TM3.

Table S1 Statistics of output sequences from the sugarcane microshoot base.

Table S2 Assembly state information of unigenes.

Table S3 Functional annotation of unigenes in different annotation databases.

Table S4 Number of unigenes in the GO terms.

Table S5 KEGG pathways of unigenes.

Table S6 DEGs in CK3 vs. TM3.

Table S7 DEGs in CK7 vs. TM7.

Table S8 Top GO terms for biological process in CK3 vs. TM3.

Table S9 Top GO terms for molecular function in CK3 vs. TM3.

Table S10 Top GO terms for cellular component in CK3 vs. TM3.

Table S11 Enriched GO terms in CK3 vs. TM3.

Table S12 Top GO terms for biological process in CK7 vs. TM7.

Table S13 Top GO terms for molecular function in CK7 vs. TM7.

Table S14 Top GO terms for cellular component in CK7 vs. TM7.

Table S15 Enriched GO terms in CK7 vs. TM7.

Table S16 Enriched KEGG pathways in CK3 vs. TM3.

Table S17 Enriched KEGG pathways in CK7 vs. TM7.

Table S18 DEGs in heatmaps.

Table S19 Primers used for qRT-PCR.
