# Supplementary figures and images for: Transcriptome Profiling Provides Molecular Insights into Auxin-Induced Adventitious Root Formation in Sugarcane (Saccharum spp. Interspecific Hybrids) Microshoots
Source: Plants (Basel). 2020 Jul 23;9(8):931. doi: 10.3390/plants9080931 (PMC7465322; doi:10.3390/plants9080931)

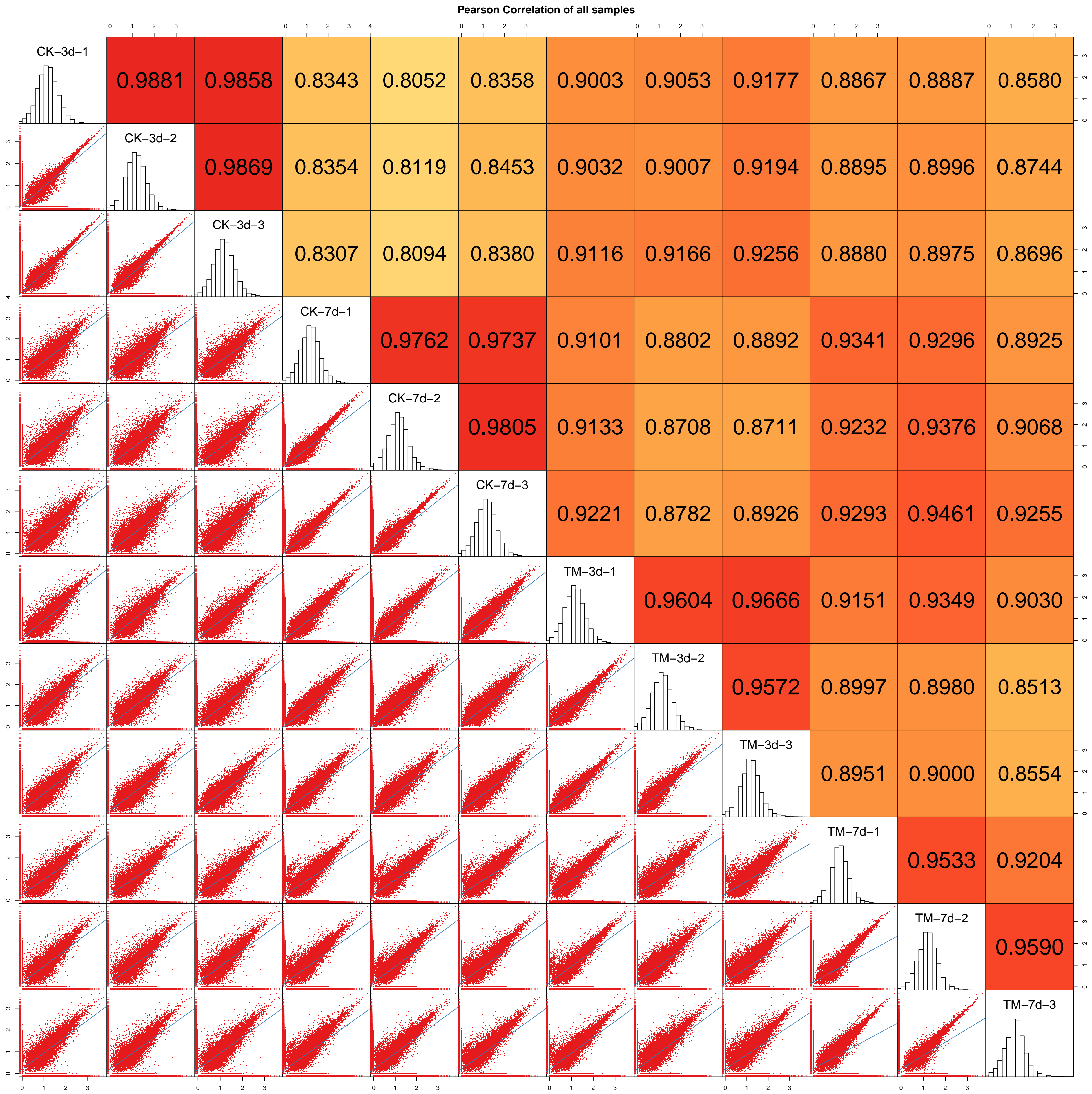

Supplement: Supplementary file 1 [file plants-09-00931-s001.zip › plants-836632-supplementary-proof/0721plants additional files/Fig S1 all_sample_cor.pdf]

Length distribution

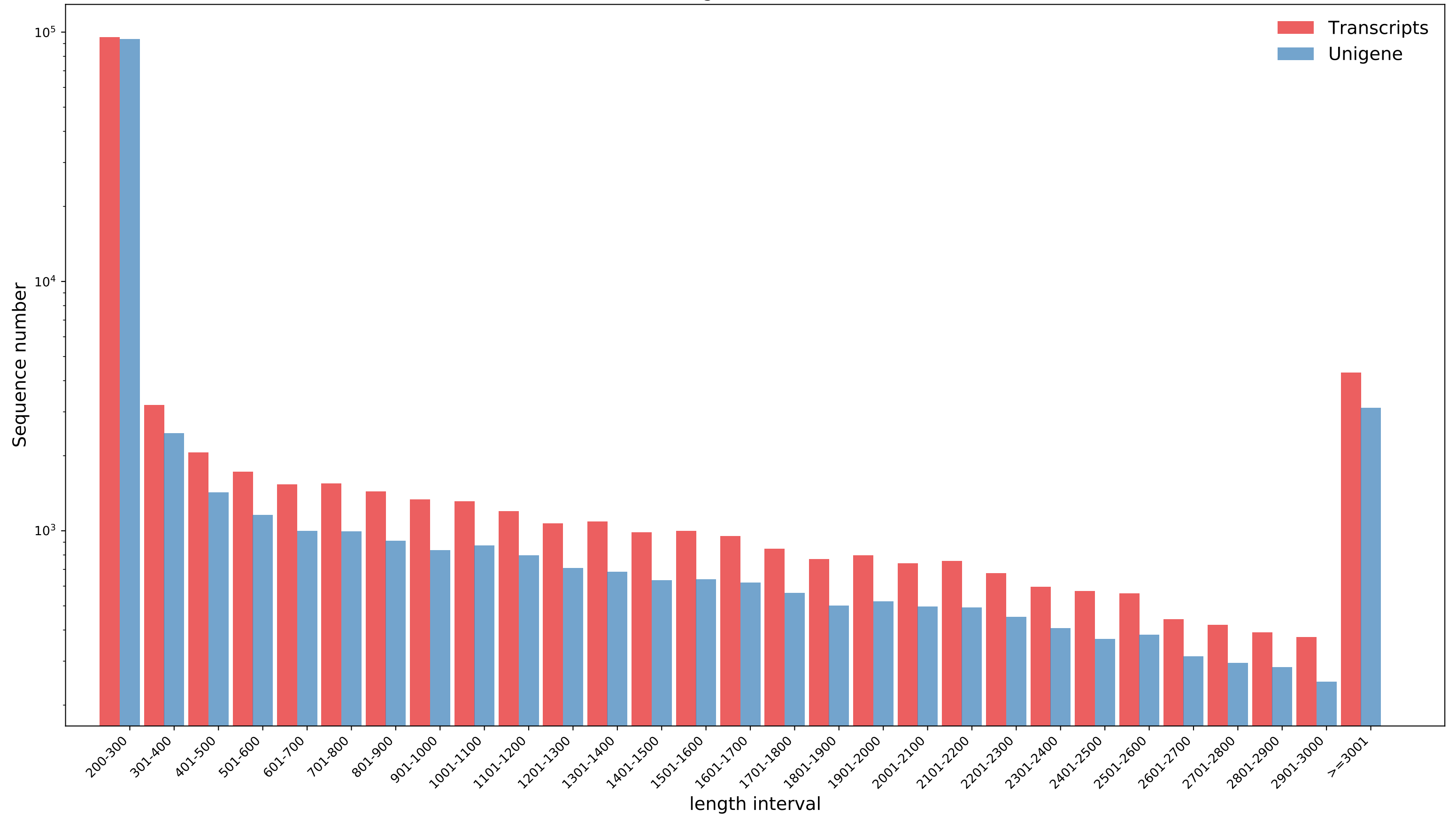

Supplement: Supplementary file 1 [file plants-09-00931-s001.zip › plants-836632-supplementary-proof/0721plants additional files/Fig S2 Assemble_length_stat.pdf]

Nr Homologous Species Distribution

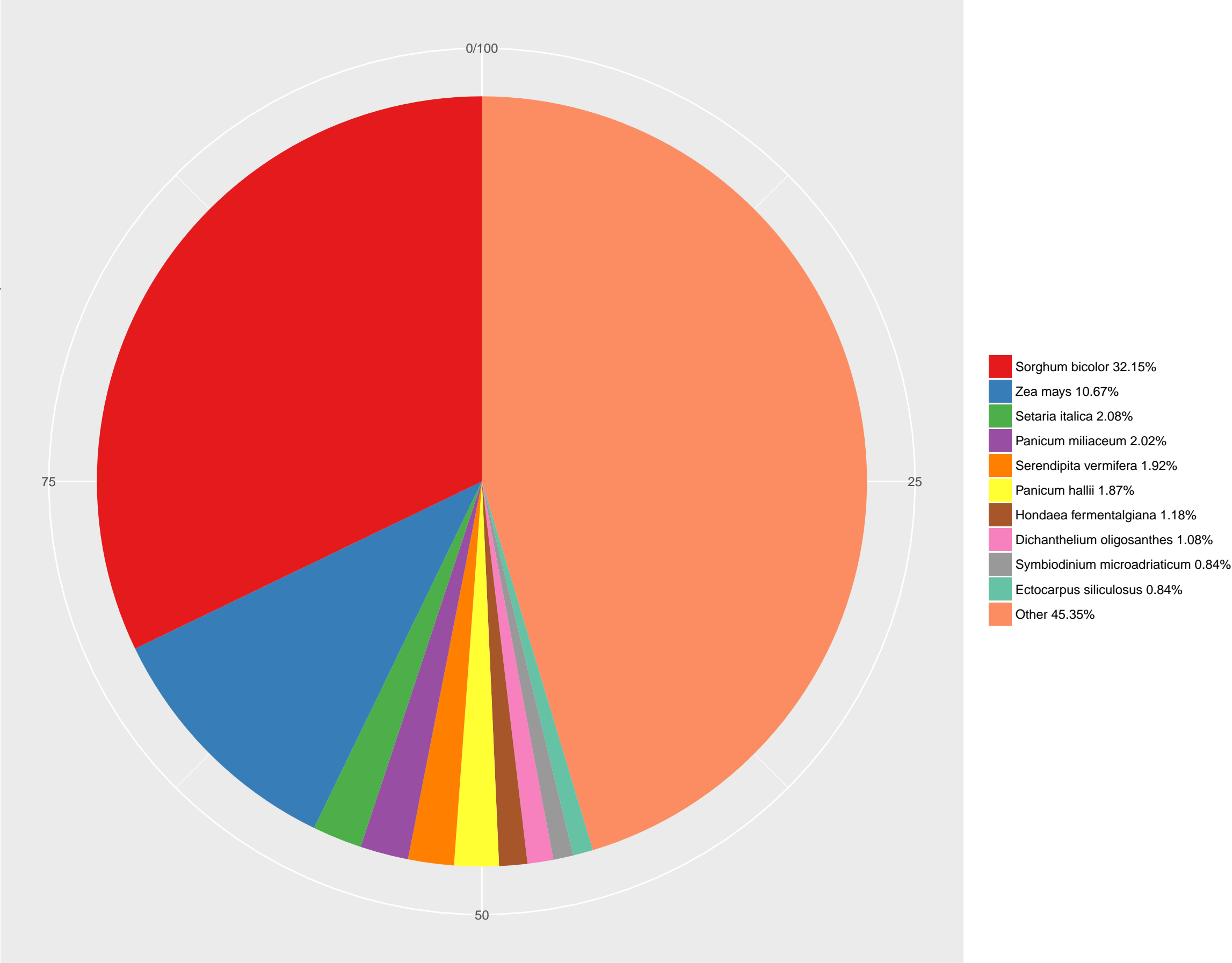

Supplement: Supplementary file 1 [file plants-09-00931-s001.zip › plants-836632-supplementary-proof/0721plants additional files/Fig S3 unigene.fa.nr.lib.pdf]

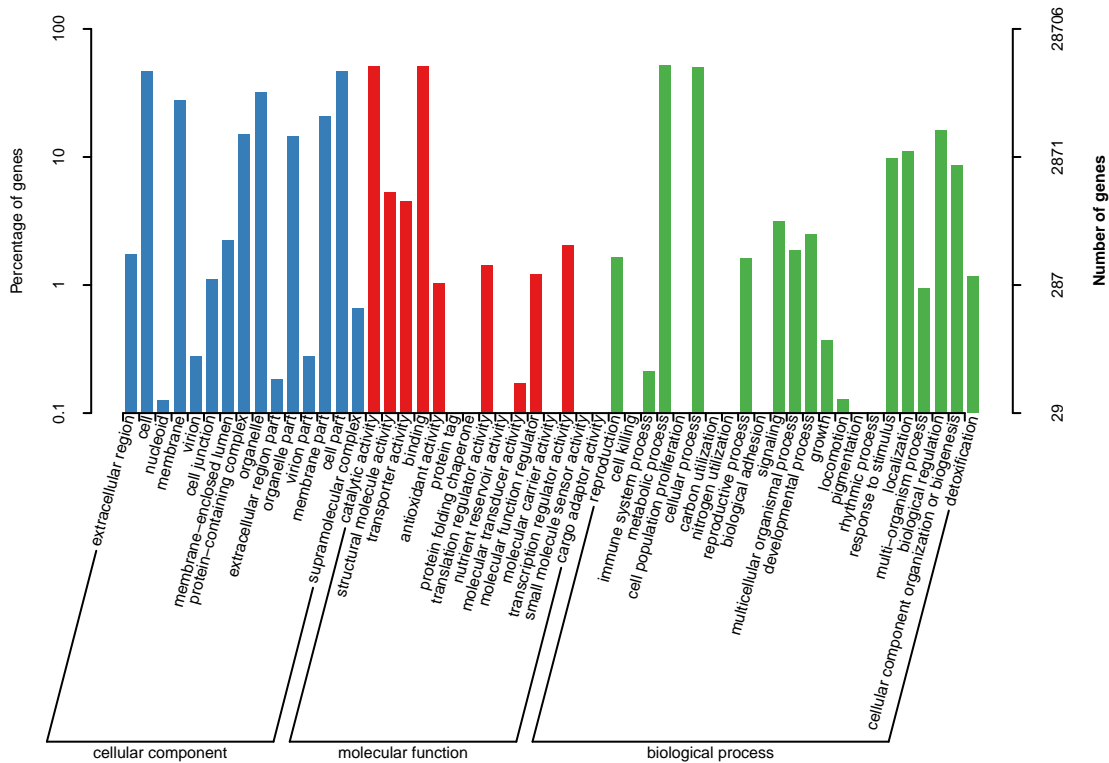

Supplement: Supplementary file 1 [file plants-09-00931-s001.zip › plants-836632-supplementary-proof/0721plants additional files/Fig S4unigene.fa.GO.pdf]

# COG Function Classification of Consensus Sequence

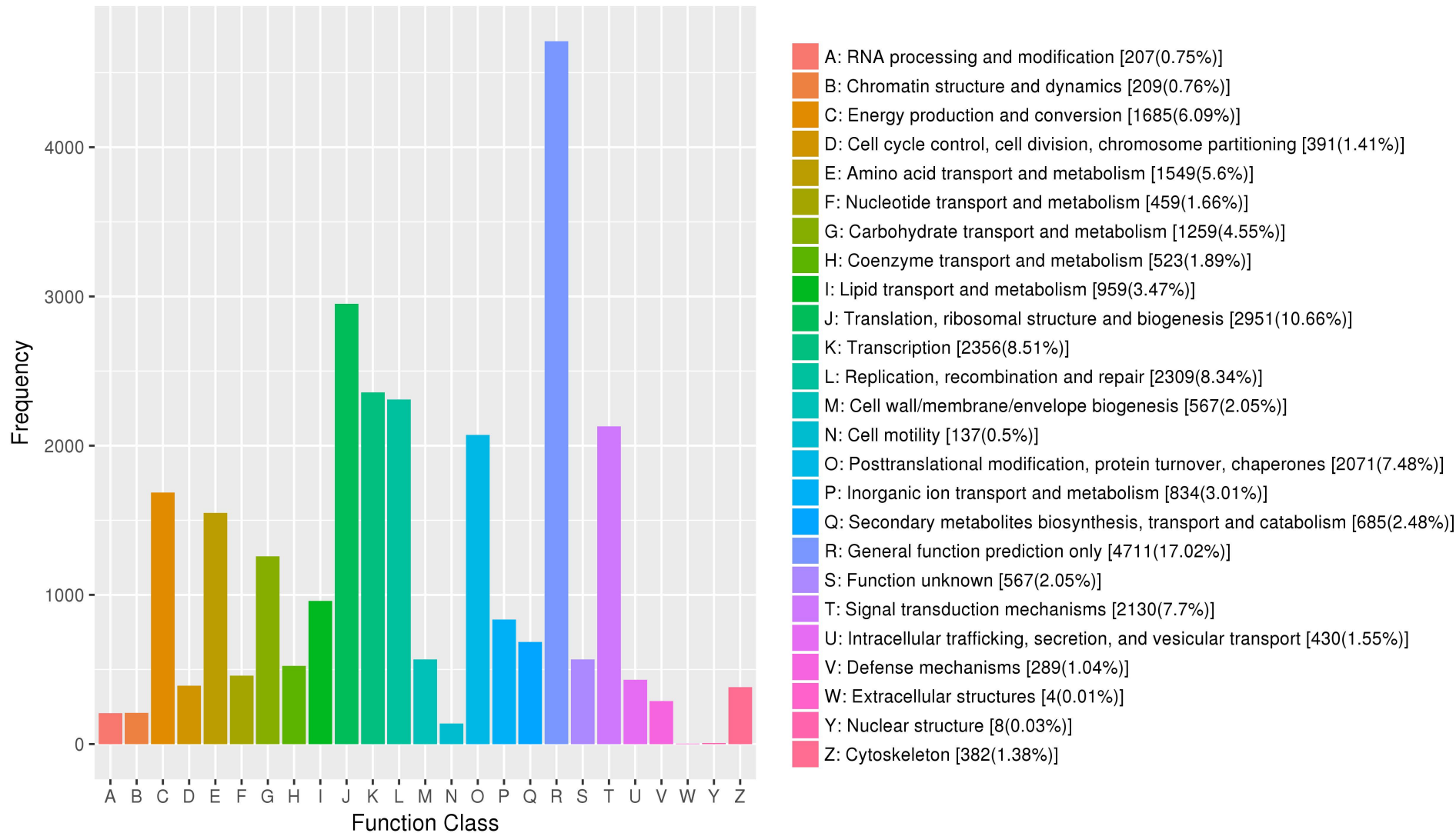

Supplement: Supplementary file 1 [file plants-09-00931-s001.zip › plants-836632-supplementary-proof/0721plants additional files/Fig S5 unigene.fa.Cog.cluster.pdf]

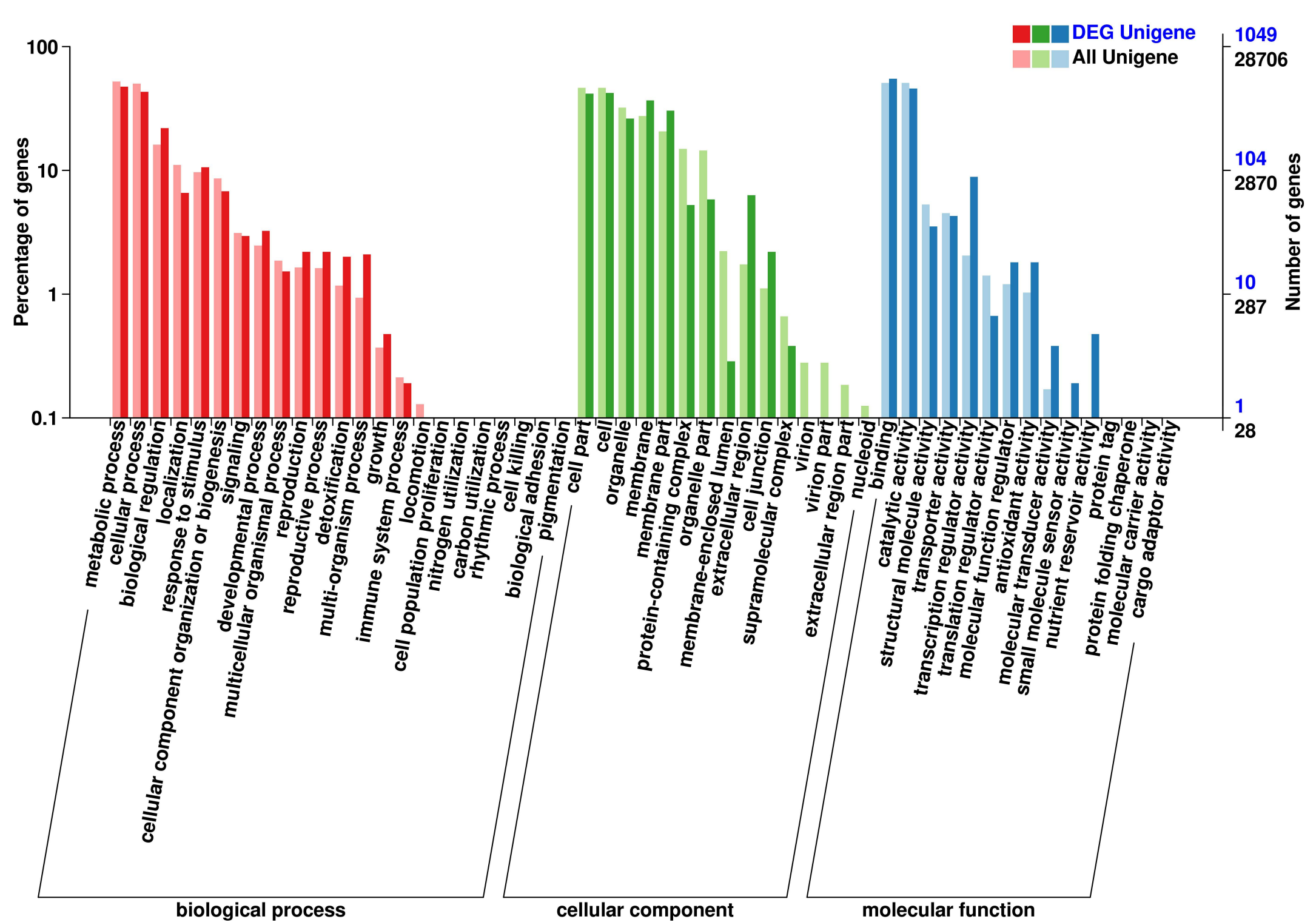

Supplement: Supplementary file 1 [file plants-09-00931-s001.zip › plants-836632-supplementary-proof/0721plants additional files/Fig S6 CK-3d_vs_TM-3d.GO.pdf]

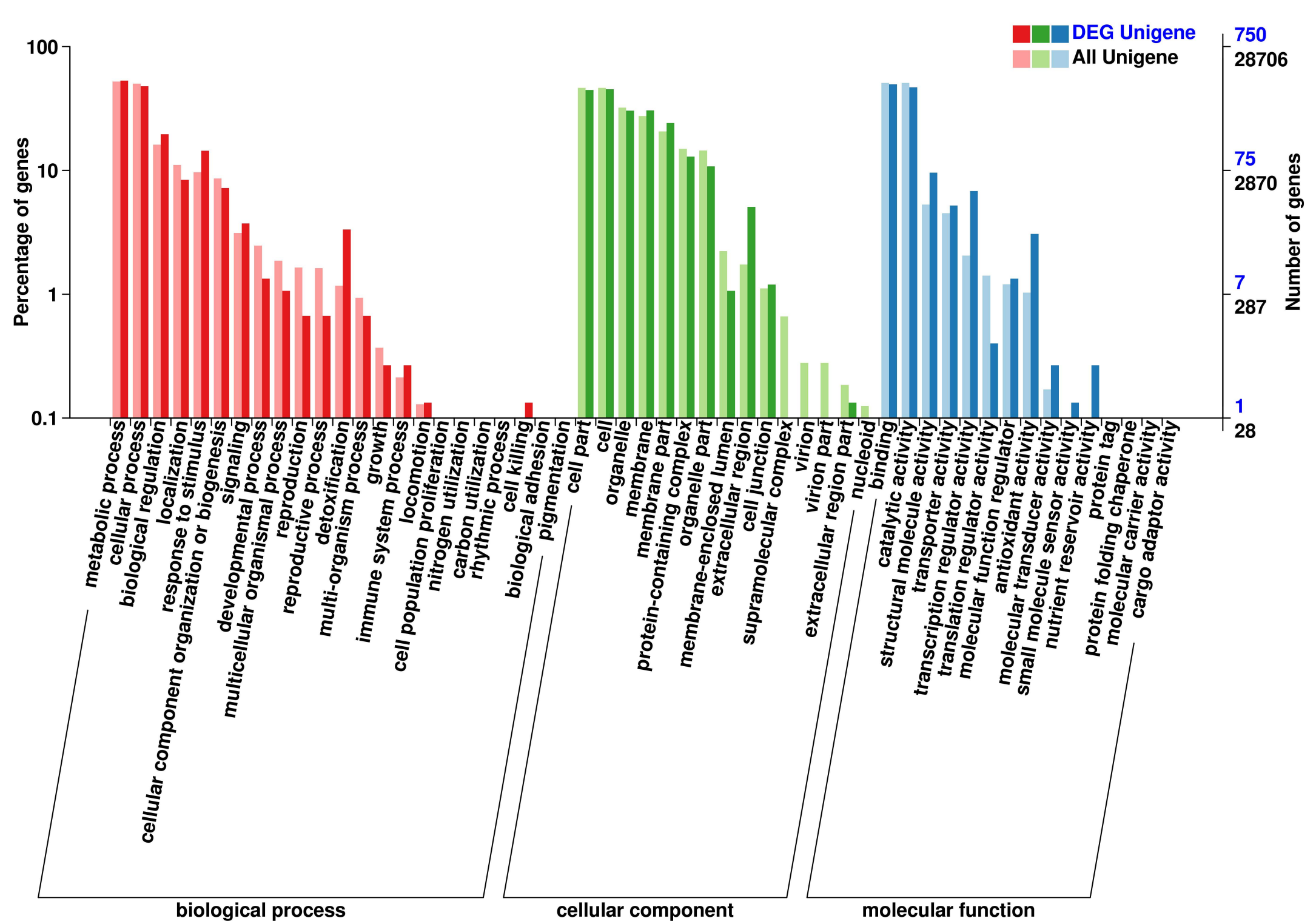

Supplement: Supplementary file 1 [file plants-09-00931-s001.zip › plants-836632-supplementary-proof/0721plants additional files/Fig S7 CK-7d_vs_TM-7d.GO.pdf]
